# LIM Kinases, Promising but Reluctant Therapeutic Targets: Chemistry and Preclinical Validation In Vivo

**DOI:** 10.3390/cells11132090

**Published:** 2022-06-30

**Authors:** Rayan Berabez, Sylvain Routier, Hélène Bénédetti, Karen Plé, Béatrice Vallée

**Affiliations:** 1 Institut de Chimie Organique et Analytique, University of Orléans, CNRS UMR 7311, CEDEX 2, 45067 Orléans, France; rayan.berabez@univ-orleans.fr (R.B.); sylvain.routier@univ-orleans.fr (S.R.); 2 Centre de Biophysique Moléculaire, UPR 4301, CNRS, University of Orléans and INSERM, CEDEX 2, 45071 Orléans, France; helene.benedetti@cnrs.fr

**Keywords:** LIMK, medicinal chemistry, in vivo preclinical validation

## Abstract

LIM Kinases are important actors in the regulation of cytoskeleton dynamics by controlling microtubule and actin filament turnover. The signaling pathways involving LIM kinases for actin filament remodeling are well established. They are downstream effectors of small G proteins of the Rho-GTPases family and have become promising targets for the treatment of several major diseases because of their position at the lower end of these signaling cascades. Cofilin, which depolymerizes actin filaments, is the best-known substrate of these enzymes. The phosphorylation of cofilin to its inactive form by LIM kinases avoids actin filament depolymerization. The balance between phosphorylated and non-phosphorylated cofilin is thought to play an important role in tumor cell invasion and metastasis. Since 2006, many small molecules have been developed for LIMK inhibition, and in this review article, we will discuss the structure–activity relationships of the few inhibitor families that have been tested in vivo on different pathological models.

## 1. Introduction

**Figure 1:**
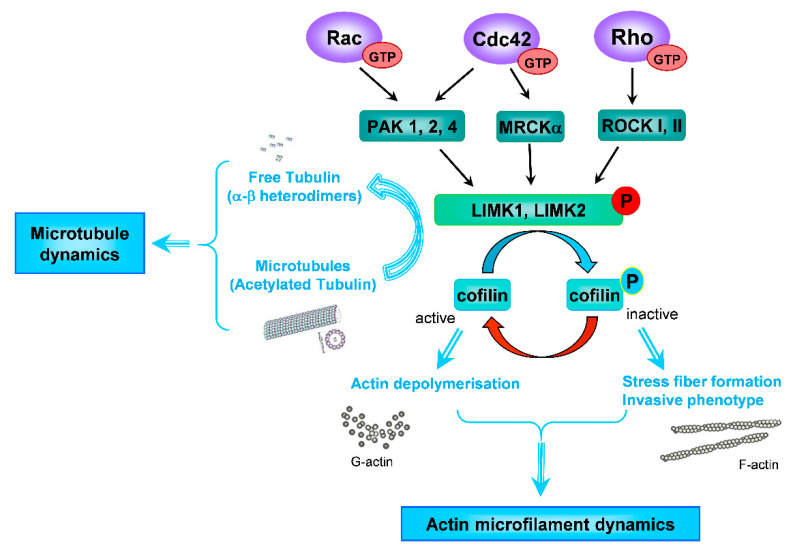
LIM Kinase signaling pathway.

The LIM kinases LIMK1 and LIMK2 (LIMKs) are serine/threonine and tyrosine kinases that play a major role in dynamic cytoskeleton regulation. They are downstream effectors of small G proteins of the Rho-GTPases superfamily, including the following cell transduction signaling pathways (Figure 1): RhoA/ROCK (Rho associated protein kinase), cdc42/PAK (p21 protein-activated kinase), Cdc42/MRCKα (myotonic dystrophy kinase-related Cdc42-binding kinase α), and Rac1/PAK, which lead to their phosphorylation and subsequent activation [1]. Activated LIMKs phosphorylate cofilin, resulting in its inhibition. LIMKs also control microtubule remodeling, independently from actin filament turnover, but the molecular mechanism of this second process remains unknown.

By regulating cytoskeleton dynamics [2,3], LIMKs are involved in numerous cellular functions, such as cell motility, morphogenesis, division, differentiation, apoptosis, neuronal morphology, and neuritogenesis. As a result, they are implicated in multiple pathologies: oncogenesis, by controlling tumor progression and metastasis development [4]; the resistance of cancers to chemotherapy targeting microtubules (MT) [5,6]; viral infections [7,8], ocular diseases (glaucoma) [9]; pain [10]; erectile dysfunction [11,12]; neurofibromatosis types 1 and 2 [13,14,15]; and neuronal diseases [16,17].

LIMK inhibition has appeared as a viable alternative to taxanes or alkaloids that have serious negative secondary effects, and resistance can develop in certain cancer cell lines over time. Moreover, LIMKs are atypical kinases, making them attractive targets for new therapies. They are dual kinases as they phosphorylate Serine or Threonine residues and Tyrosine residues. The catalytic site, the VI sequence of the C-lobe, is a mixture of both consensus sequences [18]. LIMKs are downstream of the pathway involving cofilin, their main described substrate, and specifically targeting them may avoid side effects due to the uncontrolled implication of other kinases. The process by which they interact and phosphorylate cofilin is also quite unusual. As LIMKs phosphorylate Ser3 of cofilin, the amino acids around this site do not play a pivotal role, as is usually the case for most kinases. Indeed, only the α5 helix of cofilin binds to a large interface of LIMKs, including the activation loop and the αF-αG loop, resulting in major conformational changes and the rock-and-poke of Ser3 to the active site for subsequent phosphorylation [19,20]. 

Due to their key role in the phosphorylation of cofilin and in cytoskeleton dynamics, LIMKs have become promising targets for the treatment of these various pathologies. However, since the discovery of the first inhibitors by Bristol–Myers–Squibb (BMS) in 2006 and 2008 [21,22], only one molecule has reached clinical trials for the treatment of glaucoma without going any further (phase 1/2a clinical trial, ClinicalTrials.gov (accessed on 13 June 2022) identifier NCT01528111) 

Since 2006, many new molecules have been conceived and synthesized with a variety of chemical scaffolds for LIMK inhibition with promising in vitro results. Three excellent review articles dealing with the rational design of LIMK inhibitors have been published by F. Manetti [1,23,24], and more recently by Bukhari and coworkers [25]. Most of the described molecules are Type I kinase inhibitors that bind to the active conformation of LIMKs in the ATP pocket. Several chemical families can be highlighted, e.g., aminothiazoles from BMS [21,22,26], and thieno-pyridines developed by J. Baell and coworkers [27,28,29,30,31], which demonstrated nanomolar activities against LIMKs. In 2009, Lexicon Pharmaceuticals introduced the pyrrolopyrimidine scaffold with excellent results, filing two patents and publishing one article [32,33]. Intense research was then seen from 2015 to 2017, with the appearance of seven new papers, and six of which contained this same scaffold [9,34,35,36,37,38,39]. More recently, one patent [40] and two articles [41,42] describing promising LIMK inhibitors were also published, thus confirming the high potential of this chemical series. 

**Figure 2:**
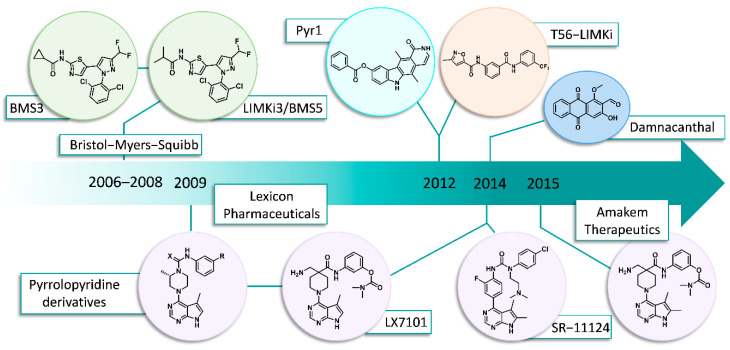
Chronological development of LIM Kinase inhibitors tested in vivo.

In this review, we have chosen to focus on the few LIM kinase inhibitors and close analogues that have reached preclinical assays in the in vivo models of different diseases, featuring, when possible, the medicinal chemistry behind these compounds (Figure 2). 

## 2. Chronology/Overview

### 2.1. Bristol-Myers Squibb (BMS)

**Figure 3:**
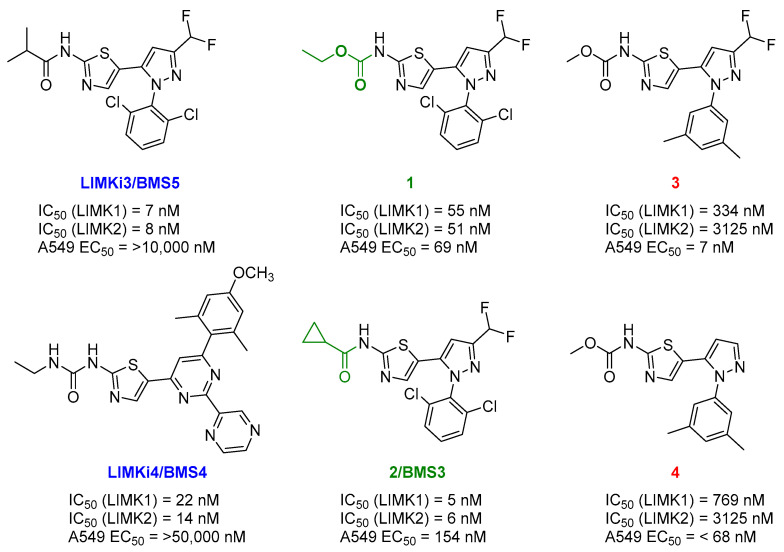
Structure and activity of BMS compounds: selective LIMK inhibitors (blue), cytotoxic LIMK inhibitors (green), and cytotoxic compounds with no LIMK activity (red).

Historically, BMS was the first pharmaceutical company to explore LIM kinase inhibition. In 2006, they published a patent of phenyl-substituted pyrimidine compounds in which more than 300 new molecules were described targeting p38 or LIMKs [21]. Although no real in vivo data was included in this patent, “test compounds” were administered orally to LPS (lipopolysaccharide) stimulated mice and the TNF-α concentration was measured in the blood samples. Two years later, they further characterized their compounds by comparing six of their lead LIMK inhibitors exhibiting different behaviors in terms of cytotoxicity and LIMK inhibition [22] (Figure 3).

One pyrimidine and five pyrazole derivatives were studied. Despite the apparent structural similarities, large differences were observed in their biological activities. The IC_50_ values described for LIMK inhibition were obtained by radioactive phosphate ATP incorporation into biotinylated full-length human destrin in the presence of the kinase domain of LIMKs overproduced in insect cells. **LIMKi3/BMS5** containing an isopropyl amide moiety showed an important inhibition of LIMK activity with an IC_50_ of 7 and 8 nM on LIMK1 and LIMK2, respectively. Surprisingly, the structurally different **LIMKi4** also presented a good inhibition of LIMKs, and both compounds were nontoxic against A549 human lung cancer cell lines. Replacing the isopropyl group of **LIMKi3** with an ethyl carbamate (**1**) or a cyclopropyl group (**2**) led to subsequent cytotoxicity (69 and 154 nM compared to >10,000 nM), while retaining LIMK inhibition. In compounds **3** and **4,** the electron withdrawing 2,6-dichlorobenzene was changed to an electron donating 3,5-dimethylbenzene substituent on the pyrazole ring while keeping a methyl carbamate moiety on the thiazole. Unexpectedly, these molecules remained cytotoxic with a net decrease in LIMK inhibition by a factor of 100 to 1000. 

The authors thus classified **LIMKi3/BMS5** and **LIMKi4** as selective inhibitors of the phosphorylation of the LIMK substrate cofilin with no cytotoxicity. Compounds **1** and **2** were described as dual compounds with an additional cytotoxicity, whereas **3** and **4** were qualified as purely cytotoxic inhibitors due to their weak inhibition of LIMKs. They then concluded that the cytotoxic compounds were targeting other proteins. They showed that these compounds (**1**, **2**, **5,** and **6**) induced mitotic arrest, a decreased labelling of tubulin in the A549 lung cell line, and the reduced mRNA levels of several tubulins. They also inhibited tubulin polymerization in an in vitro assay. The group of potent LIMK inhibitors (**3** and **4**), structurally similar to **LIMKi3/BMS5**, had no effect on cell proliferation, cell cycle turnover, tubulin labelling, in vitro tubulin polymerization, or tubulin mRNA level [22]. 

Further medicinal chemistry optimization was published in 2012 in an attempt to increase the selectivity in LIMK1 vs. LIMK2 inhibition based on a crystal structure of the complex between p38 and previously developed p38 inhibitors [26]. The IC50 values for LIMK1, LIMK2, and p38 were determined to evaluate the inhibitor efficiency and selectivity. Cofilin phosphorylation was inhibited by one of these compounds in a dose-dependent manner in HT-1080 cells (fibrosarcoma). The actin cytoskeleton was also affected by this compound, as shown via actin labelling with phalloidin.

In other studies, **BMS3** showed a promising activity on cancer cell lines in vitro. It reduced the P-cofilin level in mouse and human breast cancer cells (4T1.2 and MDA-MB-231), inhibited the 2D and 3D proliferation of both cell lines, and caused changes in the morphology of MDA-MB-231 cells grown in a 3D culture and prevented their local invasion. However, the in vivo assays were disappointing. The IP administration of **BMS3** to 4T1.2 or MDA-MB-231 xenografted mice had no effect on primary tumor growth, and even increased metastasis in the liver, diaphragm, spleen, cecum, and stomach, although its half-time was quite high (5.6 h) and phospho-cofilin levels were reduced by 60% in the primary tumors [43].

**BMS5** was tested on fear memory processing [44]. The mice were first conditioned using a contextual fear paradigm (TR). **BMS5** was injected bilaterally (0.5 µL at a concentration of 10 mM) in the hippocampus immediately after re-exposition for 5 min to the training context, and 24 h after being conditioned. The mice were then tested 24 h and 14 days later. The **BMS5**-treated mice exhibited long-term memory deficits, as they significantly displayed lower freezing behaviors. This effect was still present two weeks after re-exposure and was dose-dependent and specific to memory reactivation. These data show that LIMK inhibition resulted in an impairment in memory reconsolidation after context re-exposure but did not affect memory acquisition or extinction.

**BMS5** was also used to study chronic pain development by Zhou and coworkers [10]. Upon spared nerve injury (SNI), LIMKs were transiently activated for 72 h, to mediate central sensitization and chronic pain development. The intrathecal administration of **BMS5** during this activation period inhibited cofilin phosphorylation, prevented central sensitization, and alleviated the development of chronic pain. The effect of **BMS5** on post-surgical pain was also tested via plantar incisions in mice. In these conditions, LIMKs were quickly activated within 30 min to 1 h after incision. A single dose of 5 µg of **BMS5**, injected intrathecally 15 min before the incision, delayed the development of chronic mechanic allodynia. This showed that the inhibition of LIMKs may be an alternative for the clinical management of chronic pain.

### 2.2. Lexicon Pharmaceuticals and Amakem Therapeutics

**Scheme 1:**
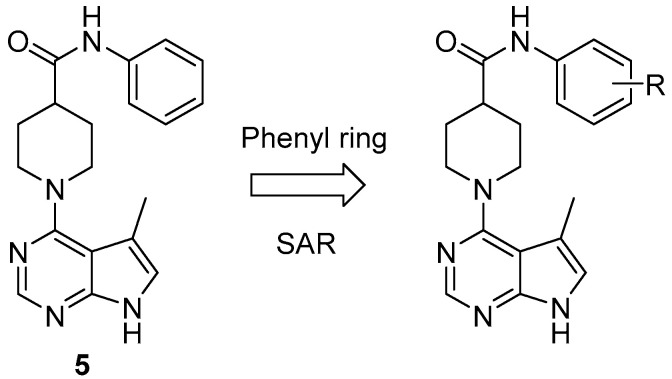
Phenyl ring Structure activity studies.

In 2009, Lexicon Pharmaceuticals targeted actin cytoskeleton dynamics for the treatment of open angle glaucoma, and discovered a novel family of LIMK inhibitors as a potent treatment for reducing IntraOcular Pressure (IOP) [33]. Since a high IOP can lead to optic nerve damage and vision loss, all the approved therapies for treating this disease are based on lowering this pressure. Indeed, relaxing the trabecular meshwork cells through the disruption of the actin cytoskeleton had been reported with several ROCK inhibitors, and a phase I clinical trial showed promising effects on IOP [45]. Unfortunately, side effects such as hyperaemia and palpebral conjunctiva were also described. In an attempt to eliminate these side effects, Lexicon Pharmaceuticals focused their attention on LIM kinases located downstream from ROCK in the pathways which affect actin regulation. Consequentially, their objectives were to develop selective LIMK2 inhibitors with a sufficient solubility in an aqueous medium for topical ocular administration. Starting from compound **5**, obtained from a high-throughput screening, the authors synthesized a series of pyrrolopyrimidines and their structure–activity relationship (SAR) highlights are shown in Scheme 1 and Table 1. 

**Scheme 2:**
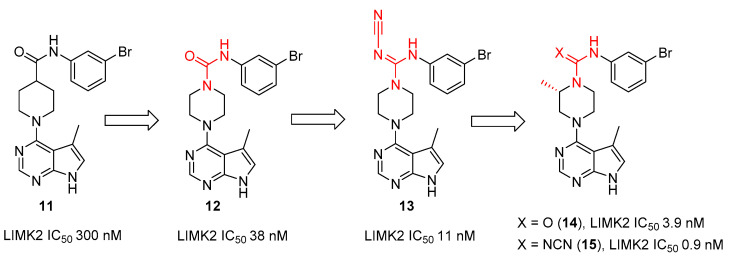
Structural optimization of Compound **11**.

First, an SAR study on the phenyl ring established that substitution in position 3 (**7**, **9**, and **10**) significantly improved the IC_50_ values as compared to positions 2 or 4, or the unsubstituted analogues (**5**, **6**, and **8**). For example, the IC_50_ of compound **8** substituted in position 4 by a methyl group was 3-fold that of the corresponding analogue **7**, and the IC_50_ increased to more than 20-fold with a substitution in position 2 (**6**). A methyl or phenyl ether (**9** and **10**) improved LIMK2 inhibition up to 64 nM, but these compounds were poorly soluble in the aqueous formulation. Compound **11** presented a promising IC_50_ value of 300 nM and a convenient solubility for in vivo assays. Further structure–activity relationships were established to improve these two characteristics. The solubility was enhanced by changing the amide function to a urea (**12**) (Scheme 2), and this modification led to an important increase in LIMK2 inhibition, with IC_50_ values reduced from 300 to 38 nM. The modification of the urea to a cyanoguanidine group **13** further improved inhibition (11 nM). 

Additionally, Harrison and coworkers introduced an (*S*)-methyl group on the piperazine ring. This substitution yielded excellent LIMK2 inhibition with IC_50_ values of 3.9 and 0.9 nM for compounds **14** and **15**, respectively. 

**Figure 4:**
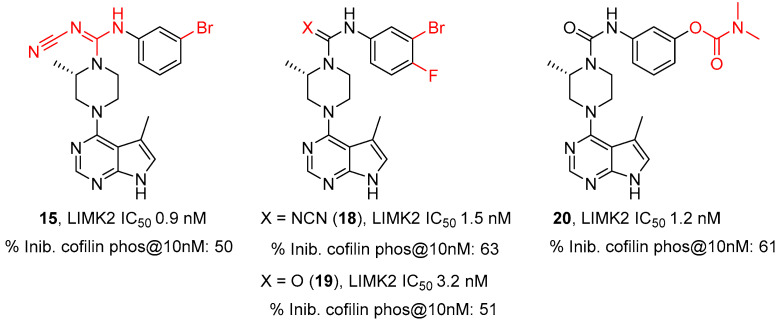
Lead LIMK2 inhibitors, Lexicon Pharmaceuticals 2009.

With the lead compounds **14** and **15** in hand, a wide range of ureas and cyanoguanides were then prepared. It was found that substituents in position 3 of the phenyl ring could be modified without dramatically affecting LIMK inhibition, including alkyl groups, carbamates, halides, amides, and sulfonamides. To further differentiate between the active compounds, they were tested for their inhibition of cofilin phosphorylation in pig trabecular meshwork cells to identify compounds exhibiting more than 50% inhibition at 10 nM. Four lead compounds (**15**, **18**–**20**) were thus identified (Figure 4).

These four compounds were further tested in vitro for LIMK1 and ROCK activity. All of them showed comparable LIMK1 and LIMK2 activities between 0.5 and 3.2 nM. The selectivity against ROCK kinases was another discriminated factor in an attempt to avoid unwanted side effects. As a result, compound **18** was eliminated with ROCK1/2 IC_50_ < 200 nM. The most effective compounds **15**, **19**, and **20** were tested in vivo on a dexamethasone-induced ocular hypertensive mouse model and formulated as a suspension in a xanthan gum-based vehicle. At a dose of 5 µg, an IOP diminution of 3.6–3.7 mmHg was observed after 1 h compared to the normal model, leading to pressure values comparable to wild type mice. As carbamate **20** showed no statistically significant results in this assay, another assay was performed that changed the dose to 1 and 0.1 mg/mL and used a co-solvent-HPMC based vehicle. These assays indicated a dose-dependent response with maximal efficacy after 1–2 h (ΔIOP = −3.1 mmHg) at a dose of 3 µg. Compounds **15** and **20** were further tested in a pig eye perfusion assay measuring inflow volume (which is directly correlated to outflow capacity). Both compounds increased the outflow capacity (30% at 100 nM), thereby corroborating previous promising data. 

The authors thus established that LIMK2 was indeed a promising target for the treatment of glaucoma. The urea **20** became the leading LIMK inhibitor for Lexicon Pharmaceuticals, with a good selectivity against ROCKs and promising in vivo results. 

**Figure 5:**
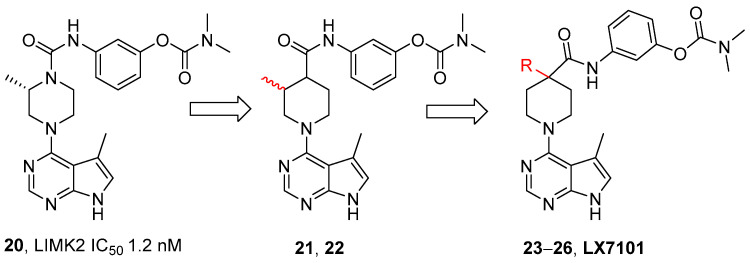
SAR optimization of compound 20.

In 2015, the same team at Lexicon Pharmaceuticals reported an improved version of their compound **20** named **LX7101** [9]. Indeed, while preparing the aqueous formulation necessary for topical ophthalmic solutions, they found that compound **20** slowly degraded over time. Further studies showed that this was mainly due to the methanol that was added for compound solubility. For example, after 24 h at 60 °C in a mixture of water/methanol 1:1, only 58% of compound **20** remained intact. The subsequent modification of the aniline portion of the molecule failed to provide more stable analogues. The authors found that the alpha substitution of the piperazine ring was the factor affecting compound stability and could be attributed to the destabilization of the planar, conjugated conformation of the piperazine nitrogen atom with the urea carbonyl, making the C−N bond susceptible to solvolysis. The replacement of the urea with an amide function yielded the necessary aqueous stability and a further SAR was performed to increase kinase inhibition (Figure 5 and Table 2).

Removing the nitrogen atom of the piperazine ring (**21**,**22**) was detrimental to activity independent of the configuration of the stereogenic center. A substitution in the alpha position relative to the carbonyl group greatly improved inhibition; with a simple allyl group (**23**), the IC_50_ dropped to 2.2 nM. The authors then decided to add solubilizing groups such as hydroxy (**24**) or tertiary amines (**25**, **26**, **LX7101**). **LX7101** was chosen as the lead compound for several reasons: it was the most soluble molecule (up to 10 mg/mL) with an excellent LIMK inhibition (4.3 and 32 nM on LIMK2 and LIMK1, respectively) and it resulted in a high IOP reduction of −4.2 mmHg after 2 h with a dose of 3 µg. The ROCK selectivity was still an issue as it was only moderately selective versus ROCK at low ATP concentrations. To mimic physiological concentrations, the authors performed the same assays with 200 µM of ATP. At this level, a greater selectivity of **LX7101** was observed against ROCK1 > 2000 nM and ROCK2 > 300 nM while keeping the IC_50_ values of LIMKs below 60 nM. It was also tested against a panel of 403 kinases, where only a moderate selectivity was observed. Binding assays on 78 different receptors showed no significant cross reactivity. 

The **LX7101** tolerability was tested on mice, rat, and rabbit eyes. It was well accepted at doses up to 0.5%. Moreover, it showed a stability of 99% in an aqueous solution formulation after 14 days at 60 °C. Its activity towards intraocular pressure in dexamethasone-induced hypertensive mice was then evaluated, and it was found to be the most effective compared to commercial glaucoma drugs (Timolo and Latanoprost), drastically lowering intraocular pressure. **LX7101** was selected for a clinical trial 1/2a to evaluate “the Safety, Tolerability, and Efficacy of LX7101 in Subjects with Primary Open-angle Glaucoma or Ocular Hypertension”. This assay started in March 2012, enrolled 63 participants, and was completed in June 2012. No results have been published (last update September 2015). The failure of this clinical trial may be attributed to the moderate selectivity of **LX7101**, as it targets other kinases and especially ROCK.

**LX7101** was further studied in triple negative breast cancer. It significantly inhibited the metastatic characteristics of MDA-MB-231 and BT-549 cell lines; the cofilin phosphorylation was decreased, the disassembly of focal adhesions and associated stress fibers was increased, and the cell migration and invasion were reduced, as well as gelatin degradation (reflecting the extracellular matrix degradation). An in vivo assay was performed using **LX7101** on breast cancer MDA-MB-231-xenografted mice. No growth inhibition of the primary tumor was observed; however, the metastasis progression was reduced [46].

**Figure 6:**
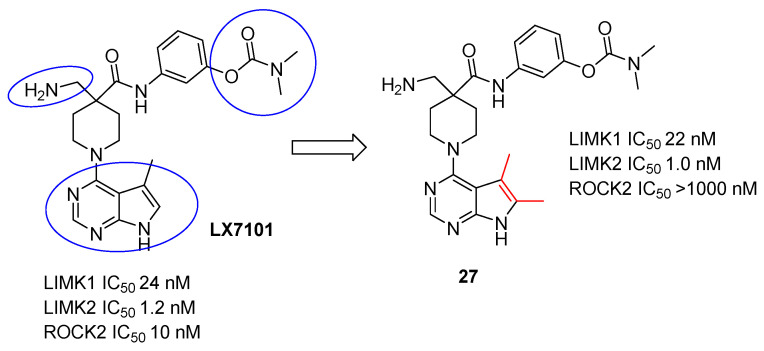
LX7101 modulation by Amakem Therapeutics.

In 2015, Amakem Therapeutics synthesized and characterized selective LIMK inhibitors based on the **LX7101** core [34]. Structural modifications were carried out on three distinct parts: the amine, the pyrrolopyrimidine scaffold, and the replacement of the carbamate moiety (Figure 6). In spite of the intensive synthetic efforts, no replacement for the pyrrolopyrimidine was found, and the best reported inhibitor possessed only an additional methyl group in the 6 position of the pyrrolopyrimidine core, which was crucial for gaining selectivity versus ROCK.

Compound **27** was demonstrated to inhibit cofilin phosphorylation, but did not affect MLC phosphorylation by MLCK, another substrate of ROCK, thus demonstrating its selectivity in contrast to **LX7101**. An in vivo assay was performed on ocular normotensive New Zealand White Rabbits, but no significant reduction of intraocular pressure was observed. However, because these rabbits have a lower IOP baseline, this could have hindered the compound’s effect. The formulation used in this assay was also different from the other studies. Nevertheless, **LX7101** was tested in the same conditions and appeared to reduce IOP. The authors thus concluded that ROCK inhibition was mainly responsible for lowering IOP rather than LIMKs, but conjunctival hyperemia was observed in some **LX7101**-treated rabbits [34].

### 2.3. SR-11124 and SR-7826: Scripps Research Institute, Florida

**Figure 7:**
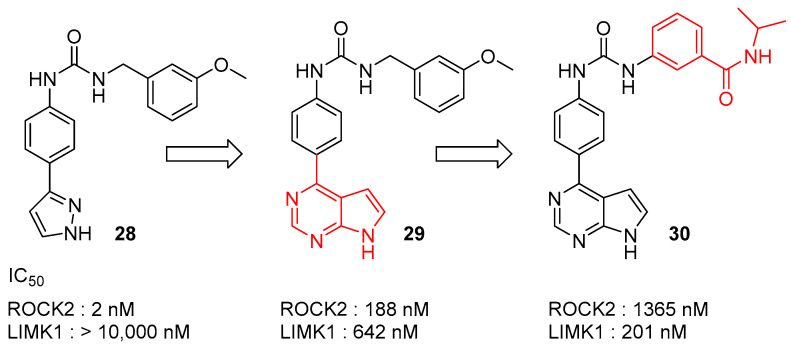
Progress toward LIMK inhibition.

In 2015, LoGrasso, Feng, and coworkers reported several bis-aryl urea derived LIMK inhibitors [38]. This interest stemmed from their previous work on ROCK inhibitors and the effect of these compounds on intraocular pressure in rats [47,48]. They were able to increase LIMK inhibition by introducing a pyrrolopyridine ring (compounds **28** and **29**, Figure 7), and then modifying the urea to yield compound **30** with an IC_50_ (LIMK1) of 201 nM that was still selective vs. ROCK. This was the beginning of their search for new and selective LIMK1 inhibitors, and an extensive medicinal chemistry program was undertaken.

SAR studies were first performed on the hinge binding region. The use of simple five- or six-membered heteroaromatic rings, such as pyrazole, pyridine, and aminopyrimidine, were effective ROCK inhibitors, with only low LIMK1 inhibition. Variation with different pyrrolopyrimidine rings yielded better selectivity and inhibition. The authors concluded that the 5-methyl and the 5, 6-dimethyl pyrrolopyrimidine derivatives were the most effective, with the latter having excellent LIMK1 vs. ROCK selectivity. 

**Figure 8:**
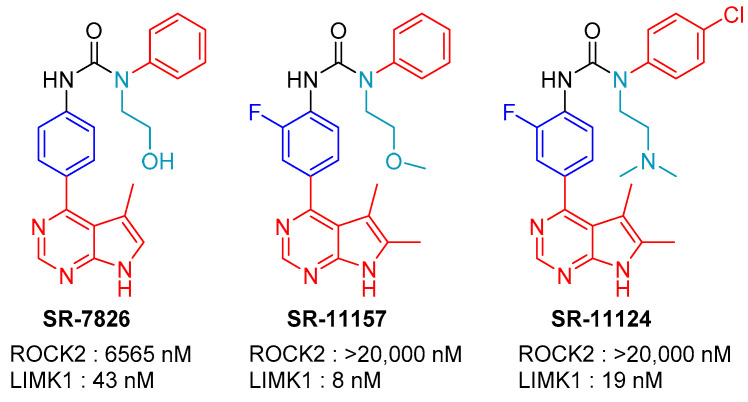
Optimized LIMK1 inhibitors.

Three substitutions on the central aromatic ring at its ortho-position (to the urea moiety), were then performed, and it was found that a fluoride atom yielded the best activity. Modification of the urea indicated that a 4-subtituent (Cl, OCH_3_, CH_3_) on the terminal phenyl group yielded the best LIMK inhibitors. The addition of a small polar group to one of the two urea NHs yielded surprising results. No substitution was permitted on the urea NH attached to the first phenyl ring, but the alkylation of the second one attached to the terminal phenyl ring improved both biochemical and cell potency, enhanced selectivity, and increased the inhibitor’s DMPK properties and bioavailability. Several optimized LIMK1 inhibitors were reported with distinct pharmacokinetic profiles, selectivity, and inhibition in various cellular tests, e.g., migration/ invasion cell-based assays, and **SR-11124** was shown to exhibit the best pharmacokinetic properties (plasma clearance and biodisponibility) (Figure 8).

To demonstrate the potential application of these LIMK inhibitors to the treatment of glaucoma, **SR-11124** was topically applied to the right eye of an elevated Intra Ocular Pressure (IOP) rat model at a dose of 50 µg. A significantly decreased IOP (>20%) was observed upon 4 h of treatment, and a high basal IOP was recovered 24 h after treatment.

In 2019, Henderson and coworkers published a study linking LIMK1 inhibition and dendritic spine resilience against β-amyloid [49]. The cognitive decline in Alzheimer’s disease (AD) results from synapse and dendritic spine loss in the brain areas critical for memory processes. Amyloid β induces the dendritic degeneration of neurons by activating the RhoA/ROCK signaling pathway. The treatment of rat hippocampal neurons with **SR-7826** resulted in a loss of Amyloid β-induced spine degeneration (in density as well as morphology). Thus, the inhibition of LIMKs downstream of ROCK could be considered as a new therapeutic possibility to treat AD. To support their hypothesis, the authors treated 6-month-old hAPPJ20 mice (models of AD) with **SR-7826** by oral gavage. No toxicity was observed, as well as no loss of weight and no liver impact after 6 h. **SR-7826** was then administrated for 11 days once a day. The global apical and basal spine densities increased in treated mice but not significantly, whereas both apical and basal thin spine densities increased significantly. Basal spine length and head diameter also increased significantly with **SR-7826** treatment. No changes in soluble/insoluble Aβ42 were observed. They also showed that the inhibition of LIMKs rescued Aβ-induced hippocampal spine loss and morphologic aberrations. Their research suggested that inhibiting LIMK1 may provide dendritic spine resilience to Aβ and could therefore benefit cognitively normal patients that are at a high risk for developing dementia.

### 2.4. Pyr1 Derivatives

Microtubules are dynamic polymers that play a key role in cell division, proliferation, signaling, and migration [50]. They are a validated target in the fight against cancer [51,52,53]. For several decades, microtubule binding agents that result in microtubules’ stabilization and subsequent cell cycle blockage have been used in cancer treatment from a diverse set of compounds such as taxanes, epothilones, vinca alkaloids, halichondrins, maytansinoids, and colchicines. However, many of these compounds have unwanted side effects, and drug resistance over time is a common cause of their failure. 

**Figure 9:**
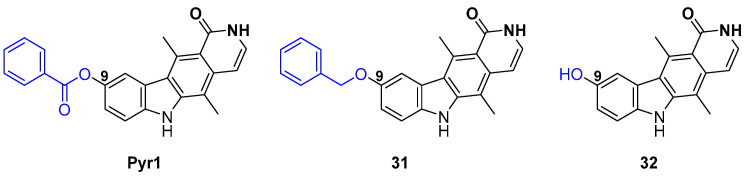
Pyr 1, *O*-benzyl derivative (**31**), and 9-OH metabolite (**32**).

In 2012, Lafanechère and coworkers focused their attention on identifying compounds that target the different proteins regulating microtubule dynamics, thus eventually leading to mitotic arrest and/or apoptosis [54]. They developed a HeLa cell-based assay that recognized detyrosinated tubulin (present in stabilized microtubules) by immunofluorescence [55]. Their screening revealed a promising candidate, the ellipticine derivative **Pyr1**, synthesized in 1983 by Rivalle and coworkers [56]. A limited medicinal chemistry SAR was performed in this series around the phenol, the central di-methyl aromatic ring, and the amide/pyridine moiety. Only eleven derivatives were reported and tested for microtubule stabilization and actin reorganization, the two most active being **Pyr1** and its corresponding benzyl derivative **31** (Figure 9) [54,57].

In their 2012 study [54], they identified LIM kinases as the main targets of **Pyr1**. In an in vitro assay, **Pyr1** was shown to inhibit the activity of recombinant LIMK1 and LIMK2 on cofilin with an IC_50_ of 50 and 75 nM, respectively. As **Pyr1** behaved as an ATP competitor, it was tested on a panel of 110 kinases, and was shown to be highly selective for LIMKs. Its toxicity was tested on different cell lines, including drug resistant ones, and a µM range of toxicity was observed. The imaging of phalloidin labelled HeLa cells revealed that **Pyr1** affected the actin microfilament cytoskeleton. It also affected microtubules, leading to their stabilization by blocking instability, and a strong reduction of the number of AB1 comets was observed upon the **Pyr1** treatment of HeLa cells. No direct interaction between **Pyr1** and tubulin was detected, which distinguished it from other MT stabilization targeting drugs. Cell cycle arrest was observed at the S to G2-M stages with an abnormal mitotic spindle and disorganized asters. MCF10A cell motility inhibition was also observed in vitro with time lapse video microscopy. The effects of **Pyr1** were also studied on these different breast cancer cell lines: TS-A-pGL3 and MDA-MB-231 [58]. In these cells, it inhibited cofilin phosphorylation, and actin dynamics were strongly diminished, with reduced filopodium-like protrusions in number and size. In a wound-healing assay, **Pyr1** was shown to reduce cell motility. A strong invasion inhibition was also detected on Matrigel transwell chambers. On highly specialized neuron cells, it was shown to decrease cofilin phosphorylation, to stabilize neuronal microtubules, and to increase total spine density [59].

**Pyr1** activity was assessed on mice models of three different diseases: leukemia, breast cancer, and schizophrenia. For each model, significant improvements were observed with no apparent toxicity (no weight loss). For leukemia, B6D2F1 female mice injected with L1210 cells were treated with 10 mg/kg daily for 10 days. The control mice were all dead 70 days after the assay began, whereas the **Pyr1**-treated mice were still all alive [54]. For breast cancer, NMRI mice were injected with TS/A-pGL3 or MDA-MB-231 cells. **Pyr1** stopped tumor growth (with a decrease of tumor volume of 40 to 50%) and decreased cellular density in the tumors. No change in the phospho-cofilin rate was observed, whereas an increase of deTyrMT (detyrosinated microtubules) was noted, suggesting a stabilization of the microtubules. **Pyr1** is thought to act on breast cancer tumor growth via MT. The metabolization of **Pyr1** into its 9-OH metabolite **32** was observed, and this compound seemed to be the active one (Figure 8). Furthermore, **Pyr1** did not affect the number of lung metastases; however, their size was reduced. When MDA-MB-231 cells were directly injected into the bloodstream of **Pyr1**-treated mice, the number of metastases was not reduced, although a significant decrease in the global metastatic load was observed, and this effect lasted several days after the end of the **Pyr1**-treatment [58]. For Schizophrenia, MAP6 KO mice were used. **Pyr1** was able to cross the Blood Brain Barrier and restored the normal dendritic spine density and morphology, and mature and functional spines were recovered. **Pyr1** also improved the long-term potentiation and synaptic plasticity. It also reduced social withdrawal (sniffing time was increased) and depressive/anxiety-like behavior (evaluated by the Novelty Suppressed Feeding test; the latency to eat was strongly reduced) [59].

### 2.5. T56-LIMKi (T5601640)

**Figure 10:**
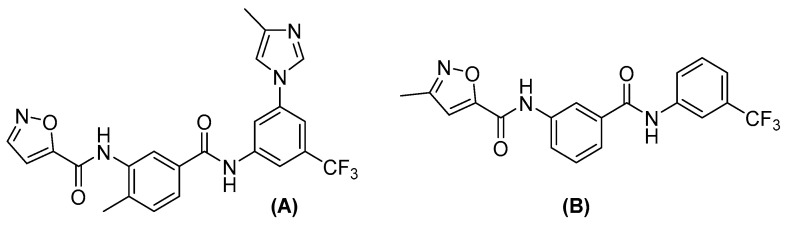
AWL-II-38.3 (**A**) and T56-LIMKi (**B**).

A traditional medicinal chemistry strategy using small molecule synthesis and structure activity relationships was not used to find the LIMK2 inhibitor **T56-LIMKi**, also known as **T5601640**. Instead, Wolfson and coworkers reported a new oxazole based inhibitor of LIMKs 1/2 through a molecular modelling approach [60]. Their quest for a new LIMK inhibitor first started with the search for proteins homologous to LIMKs, particularly LIMK2. They found that the tyrosine kinase receptor EphA3, a therapeutic target for cancer metastasis [61], exhibited a 31% sequence identity with LIMK2. Further exploration of the PDB led to a solved crystalline structure (PDB ID: 3DZQ) with the inhibitor **AWL-II-38.3** blocking the active site [62]. Bioinformatics were used to model the structure of the kinase domain of LIMK2 with the EphA3 kinase structure as a template, and the inhibitor-binding sites of the two proteins were compared. The authors found a highly conserved binding site between EphA3 and LIMK2, leading to the hypothesis that the EphA3 inhibitor might also inhibit LIMK2. Searching the ZINC database of commercially available compounds that were similar to **AWL-II-38.3** (A) led to the structurally similar **T56-LIMKi** (B) (Figure 10). The origin of this molecule remains unclear, as we found no information concerning its synthesis or the preparation of any analogues. 

The authors tested **T56-LIMKi** on *NF1*-depleted Mouse Embryonic Fibroblasts (*NF1* −/− MEF) as they had previously showed that P-cofilin levels were very high in these cell lines [14]. *NF1* −/− MEF were serum starved for 24 h, and then incubated with different amounts of the active compound. **T56-LIMKi** reduced cofilin phosphorylation in a dose-responsive manner (10–50 μM). **BMS-5** was also tested in the same conditions and appeared to be more potent. **T56-LIMKi** also affected *NF1* −/− MEF growth; its IC50 was 30 µM with a 5 day treatment. The cytoskeleton was also affected by treatment at a high concentration (50 µM) with a significant decrease of actin stress fibers visualized by phalloidin labelling as well as a decrease in cell migration observed by a wound healing assay. It also inhibited colony formation in a dose-dependent manner, as tested by an anchorage-independent growth on soft agar. It was also combined with Salirasib, a Ras inhibitor, as Ras activity is increased by the loss of NF1, which encodes for a Ras-GAP (GTPase Activating Protein) in *NF1* −/− MEF. 

Two years later, the same team further characterized **T56-LIMKi** [63]. They reported that it was highly specific for LIMK2. HeLa cells, stably expressing LIMK1 or LIMK2, were starved for 24 h and then treated with 50 µM **T56-LIMKi** for 2 h. They showed a strong and significant reduction of phospho-cofilin in the presence of LIMK2, but not in the presence of LIMK1 or in the non-transfected control cells. **T56-LIMKi** also inhibited the proliferation of various cancer cell lines in a cell specific manner [63]. The best activity was observed with glioblastoma (U87), schwannoma (ST88-14), and pancreatic cancer (Panc-1) cell lines as well as *NF1* −/− MEF (IC50 ranging from 7 to 35 µM). They also studied the level of phospho-cofilin in these cell lines after a 2 h treatment with 50 µM T56-LIMKi preceded by a 24 h starvation. The strongest decrease in phospho-cofilin was observed for Panc-1 cells with about 50% reduction (stronger than **BMS5** tested in the same conditions). Based on these results, the Panc-1 cell line was chosen to conduct an in vivo assay. First, the toxicity of **T56-LIMKi** was evaluated upon single oral administration (poor solubility limited its injection mode). No toxicity was observed for doses ranging from 20 to 100 mg/kg (no weight loss). Nude CD1-Nu mice were injected with Panc-1 cells just above the right femoral joint. When the tumor volumes reached 0.06–0.07 cm^3^ (7 days later), the mice were fed either with a vehicle (0.5% CarboxyMethylCellulose, CMC), 30 mg/kg or 60 mg/kg of **T56-LIMKi** daily by oral administration. A dose- and time-dependent decrease in tumor volume was observed, with a stronger effect for the 60 mg/kg dose. For half of the animals, the tumors completely disappeared after a 35-day treatment. The tumors were then homogenated to analyze phospho-cofilin levels and they showed a 25% reduction for the 60 mg/kg treatment compared to the untreated animals. 

In a recently published article, Demyanenko and Uzdensky studied the possible neuroprotective effects of several protein kinase inhibitors on infarction size and morphology of the peri-infarct area in the mouse brain after a Photothrombotic Stroke (PTS) [64]. Previous proteomic studies highlighted the upregulation of more than 80 proteins in the peri-infarct area after a stroke with cofilin being one of the detected proteins. The authors focused on this particular protein, as it is ubiquitous and abundant in neurons, and plays a crucial role in neurofilament dynamics. Since no selective inhibitor of cofilin was found, they decided to use **T56-LIMKi**, which is commercially available. They treated CD-1 mice with 30 mg/kg of the active compound orally once a day for 5 days for 1 h upon PTS. **T56-LIMKi** was found to reduce the infarction volume by 2 or 3.4 times at 7 or 14 days after PTS, respectively. It also rescued morphological changes in the brain (pericellular edema and conversion of nervous interconnective tissue) and reduced the number of pathologically altered cells (pyknotic, hypo-chromic, and hyperchromic cells). Two other kinases were inhibited in a similar assay, but no effects were observed. Thus, **T56-LIMKi** was shown to be a neuroprotective agent through its protection of the mouse brain from the negative consequences of PTS. LIMK2 may be considered as a potential target for anti-ischemic therapy.

### 2.6. Damnacanthal 

**Figure 11:**
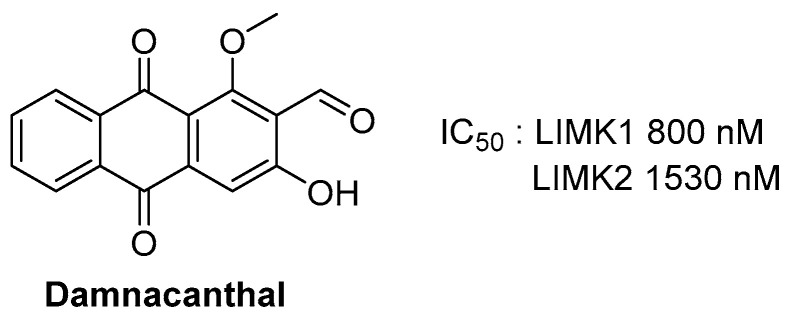
**Damnacanthal**.

**Damnacanthal** (Figure 11) is a natural product extracted from the roots of a tropical plant native to Thailand, *Morinda citrifolia* [65]. It was discovered as a LIMK1 inhibitor during screening with the bimolecular fluorescence complementation (BiFC) assay system to detect the interaction between G-actin and cofilin [66]. 

Its specificity was assessed on a panel of eight kinases, and it was shown to inhibit LIMK1 (IC_50_ 0.80 µM), LIMK2 (IC_50_ 1.53 µM), and Lck (IC_50_ 1.62 µM), but not ROCK, PAK3, PKCα, or CaMIKα (IC_50_ > 20 µM). To discriminate between LIMK and Lck activity, it was tested it on several cell lines expressing different levels of Lck. N1E-115 cells transfected with LIMK1 and treated with **Damnacanthal** exhibited a lower amount of phospho-cofilin and a reduced deceleration of actin retrograde flow. It was also shown to inhibit the CXCL12 (SDF-1)-induced chemotactic migration of Jurkat lymphocyte T-cells and Lck-deficient JCaM1.6 cells. **Damnacanthal** activity was also tested on human breast carcinoma cells, MDA-MB-231, which exhibit no expression of Lck. It was shown to inhibit the migration and the invasion of the cell line upon serum-induction.

Finally, to further test the activity of **Damnacanthal** on an in vivo model, it was tested on epidermal Langerhans cells in mouse ears. These cells are dendritic cells that initiate the cutaneous immune response by migrating from the epidermis to draining lymph nodes in response to chemical allergens on the skin surface to trigger an immune response. To examine whether **Damnacanthal** affected cell migration in vivo, it was topically administrated at 20 µM on mouse ear skin 30 min before, immediately after, and 12 h after the application of 2,4,6-trinitrochlorobenzene (TNCB). The density of Langerhans cells in the ear epidermis was much higher for the **Damnacanthal**-treated animals. These results thus suggested that LIMK inhibition suppresses the hapten-induced migration of epidermal Langerhans cells in mouse ears. 

### 2.7. CEL_Amide

Acute Myeloid Leukemia (AML) is a heterogeneous group of diseases. One AML disease, induced by mutations in FLT3-ITD, has been implicated in chemoresistance. Several FLT3-ITP inhibitors exist but have only limited action. ROCK has been shown to be constitutively activated when FLT3-ITD is mutated. The pharmacological inhibition of ROCK has thus shown promising results on FLT3-ITD mutated cell lines. When ROCK is activated, the downstream LIM kinases inactivate cofilin via phosphorylation. When LIMK is inhibited, cofilin is activated via dephosphorylation, and it localizes to mitochondria and induces apoptosis, making LIM kinases attractive targets for reducing chemoresistance in FLT3-ITD mutated patients.

In a recent paper, Braun and coworkers investigated the preclinical effects of the LIMK1/2 inhibitor **CEL_Amide** in FLT3-ITD mutated Acute Myeloid Leukemia cells [67]. The authors targeted LIMKs by using **CEL_Amide** (LIMKi), a LIMK inhibitor unrelated to the BMS compounds **LIMKi3** and **LIMKi5**. This compound was provided by Cellipse, the start-up company funded by **Pyr1’s** discovery. No chemical structure was published in the article, but a molecular weight of 632.1 g.mol^−1^ was given for this molecule as its sodium salt. The kinase selectivity of this compound was performed on a panel of 58 recombinant protein kinases, and it showed significant kinase activity reduction towards LIMK1 and LIMK2, but also on the A-Raf, B-Raf, C-Raf, cKIT, FLT1, FLT4, Ret, and Src kinases. No significant toxicity towards CD34+ cells from healthy donors was observed even at high doses (up to 1500 nM), whereas submicromolar IC_50_ values were determined on different AML cell lines. LIMK1 or LIMK2 expression was shown to be reduced in some of these cell lines upon treatment with **CEL_Amide**, as well as cofilin phosphorylation.

In vivo tests were performed on NOD-SCID mice engrafted with a MOLM13-LUC cell line (FLT3-ITD AML). From day 11, and once a day for 3 weeks, the mice were treated either with the vehicle, 10 mg/kg of CEL-Amide I.V., or midostaurin 100 mg/kg p.o., or a combination of both. **CEL_Amide** alone had no effect. The combination of **CEL_Amide** and midostaurin delayed MOLM-13-LUC engraftment and significantly prolonged mouse survival compared to midostaurin treatment alone.

Table 3 provides a brief summary of the in vivo conditions, observations and results that were obtained for the different molecules discussed in this article.

## 3. Conclusions

Throughout this review, we have seen that LIM kinases are indeed promising but reluctant therapeutic targets. Due to their implication in cell dynamics and migration, as well as their position as downstream effectors of several signalization pathways, the search for potent and selective LIM kinase inhibitors has been ongoing since the early reports of BMS in 2006. Different chemical series have been developed that target a wide number of diseases. Improvements have been made in selectivity with regard to upstream kinases and in targeting LIMK1 vs. LIMK2, as well as in designing compounds with improved pharmacokinetic properties. Despite this intensive research, there have only been nine molecules described in in vivo assays, and only one known to have advanced to a clinical trial. It is clear that the design of a unique chemical series meeting the multiple criteria of both targets and exhibiting specific activity on specialized cells by structural modulation, combined with excellent ADME parameters, has not yet been achieved. 

A potential answer to these problems could lie in the design and synthesis of LIMK specific PROTACs (Proteolysis-targeting chimeras). Although this strategy has been widely applied to different “undruggable” protein targets [68], relatively few papers have explicitly dealt with kinase degradation [69]. In the case of LIMK inhibition, a clear advantage is that instead of only targeting the active site to decrease or totally inhibit enzyme activity, a LIMK specific PROTAC would lead to the ubiquitination and degradation of the protein in the cell. In a recent paper, Fischer and coworkers classified LIMKs as being highly degradable targets using this approach [70]. Our laboratory is currently involved in the preparation of these types of compounds.

It is thus clear that more work is necessary to find promising candidates that are effective in vivo and that have the potential to continue into clinical trials, making the development of new inhibitors an exciting and on-going challenge to be met. 

## Figures and Tables

**Table 1:** Phenyl ring Substitution.

Compd	R	LIMK2 IC_50_ (nM)
**5**	H	800
**6**	2-Me	11,500
**7**	3-Me	650
**8**	4-Me	1300
**9**	3-OMe	520
**10**	3-Oph	64
**11**	3-Br	300

**Table 2:** Water-stable amides and in vitro/in vivo assays.

Compd		R	LIMK2	LIMK1	ROCK1	ROCK2
			IC_50_ (nM) ^a^			
**21**	Me (trans)	--	210	--	--	--
**22**	Me (cis)	--	110	--	--	--
**23**	--	Allyl	2.2	--	--	--
**24**	--	OH	138	--	--	--
**25**	--	NMe_2_	5.1	--	--	--
**26**	--	CH_2_CH_2_NMe_2_	7.3	134	139	ND
**LX7101**	--	CH_2_NMe_2_	4.3 (7.5)	32 (54)	69 (2200)	32 (340)

^a^ IC_50_ values at 2 µM ATP, in parentheses at 200 µM ATP.

**Table 3:** In vivo LIMK inhibitors.

Molecule	Disease	Animal Model	Treatment Procedure	Observations and Results	Ref.
BMS3	Breast cancer	Balb/c or NSG mice with 4T1.2 cells injected into the mammary gland	- 20 control, 20 treated - 20 mg/kg in 10% DMSO/0.5% soln. in saline - IP daily for 30 days (day after cell injection)	- Still detectable in plasma 24 h after injection, half time of 5.6 h - Reduced level of phospho-cofilin - No effect on primary tumor growth - More numerous and larger tumor nodules in the lung	Li et al., 2013 [43]
	Breast cancer	SCID mice with MDA-MB-23 cells injected into the mammary gland	- 5 control, 5 treated - 20 mg/kg in 10% DMSO/0.5% soln. in saline - IP daily for 29 days	- Reduced phospho-cofilin (60%) - Slight lag in tumor growth	Li et al., 2013 [43]
BMS5-LIMKi3	Memory reconsolidation	C57BL/6 male mice conditioned using a contextual fear paradigm, 24 h later were re-exposed to the training context for 5 min	- 10 mM (0.5 µL) bilateral injection in the hippocampus before the training session	- Memory reconsolidation impairment after context re-exposure	Medina et al., 2020 [44]
	Chronic pain	C57BL/6 intraplantar injection of saline/CFA (complete Freund’s adjuvant) 1/1	- 2, 5, and 10 µg intrathecal injection	- Alleviation of chronic pain development	Yang et al., 2017 [10]
Lexicon	Glaucoma	Dexamethasone-induced ocular hypertensive mouse perfused pig eye	- 5 µL of 1 mg/mL xanthan gum suspension of compound - Infusion of 100 nM soln. at constant pressure	- Reduced increased intraocular pressure to normal level - Increased outflow capacities	Harrison et al., 2009 [33]
LX7101	Glaucoma ocular hypertension	Dexamethasone-induced ocular hypertensive mouse clinical trial phase I	- 3 µL of 5 mg/mL HPMC based aqueous soln.	- 99% stability in an aqueous soln. after 14 days at 60 °C - Reduced increased of intraocular pressure	Harrison et al., 2015 [9]
LX7101	Breast cancer	NSG mice injected with MDA-MB-231	- 4 mice - 5 mg/kg in 0.5% methyl cellulose in PBS - Oral gavage for 4 weeks every other day 4 weeks after cell injection	- No inhibition of primary tumor growth - Reduced metastatic progression	Malvi et al., 2020 [46]
Compound 27 Amakem	Ocular hypertension	Ocular normotensive New Zealand White Rabbit	- 5 animals - 0.5% *w*/*v* soln. in PEG 400/water 1/1 - 40 µL eye drop (200 µg/eye drop)	- No significant reduction of intraocular pressure	Boland et al., 2015 [34]
SR11124	Ocular hypertension	Old brown Norway rats initial elevated Intraocular Pressure (IOP) of 28 mm Hg	- 6 rats left eye control, right eye treated) - 50 µg applied once - Topical admin.	- Reduced intraocular pressure in rat eyes 4 h upon treatment	Yin et al., 2015 [38]
SR7826	Alzheimer’s disease	hAPPJ20 mice	- 5 control, 5 treated - 10 mg/kg in 90% H_2_O/10% DMSO - Oral admin. daily for 11 days	- Rescued Aβ-induced hippocampal spine loss and morphological aberrations	Henderson et al., 2019 [49]
Pyr1	Leukemia	B6D2F1 female mice were injected subcutaneously in the right flank with 2 × 10^6^ L1210 cells	- 6 control, 6 treated - 10 mg/kg/d in PEG 400 - Intraperitoneally, on the left flank, daily for 10 days	- No apparent toxicity - 100% vehicle-treated mice dead at day 70 - 100% Pyr1-treated mice alive at day 90 => complete survival gain	Prudent et al., 2012 [54]
	Breast cancer	NMRI nude mice with TS/A-pGL3 cells stably transfected with luciferase injected into the mammary fat pad	- 10 control, 10 treated - 10 mg/kg in 36% PEG 400, 10% DMSO, 54% NaCl 0.9% - IP daily for 14 days (7 days after cell injection)	- Tumor followed by bioluminescence - Pyr1 metabolization into 9-OH metabolite M1, which disappeared within 2 h - In the tumor: acetyl and detyrosinated tubulin increase, phospho-cofilin unaffected - Pyr1 stopped tumor growth	Prunier et al., 2016 [58]
	Breast cancer	NMRI nude mice with MDA-MB-231 injected into the right flank	- 10 control, 10 treated - 10 mg/kg in 36% PEG 400, 10% DMSO, 54% NaCl 0.9% - IP daily for 7 days (21 days after cell injection)	In the tumor: significant decrease in volume, reduced cellular density, acetyl and detyrosinated tubulin increase, phospho-cofilin unaffected	Prunier et al., 2016 [58]
	Breast cancer	NSG female injected in the mammary fat pad with MDA-MB-231 Dendra2	- 3 control, 4 treated - 10 mg/kg in 36% PEG 400, 10% DMSO, 54% NaCl 0.9% - IP daily for 14 days (30 to 45 after cell injection)	- In the tumor: cells were rounder and less packed, cell proliferation was decreased, apoptotic cells were more numerous - Rounder cells had higher velocity compared to elongated ones - No effect on the number of lung metastases, but sizes were reduced	Prunier et al., 2016 [58]
	Breast cancer	NMRI nude mice with MDA-MB-231-ZNF217rvLuc2 stably transfected with luciferase injected into the left ventricle	- 9 control, 15 treated - 10 mg/kg in 36% PEG 400, 10% DMSO, 54% NaCl 0.9%—IP daily for 35 days (3 days before cell injection)	- No effect on the number of metastases - Strong reduction of metastatic growth	Prunier et al., 2016 [58]
	Schizophrenia	C57BL6/129SvPas-F1 MAP6 KO male mice	- 100 mg/kg/week in 0.43% NaCl, 32% PEG 400 - IP twice a week for at least 6 weeks	- Restored normal dendritic spine density - Improved long-term potentiation - Improved long-term synaptic plasticity - Reduced social withdrawal and depressive/anxiety-like behavior	Gory-Fauré et al., 2021 [59]
T56-LIMKi	Pancreatic cancer	Nude CD1-Nu mice injected with 5 × 10^6^ Panc-1 cells	- 8 control, 8 treated - 30 or 60 mg/kg in 0.5% CarboxyMethylCellulose: CMC - Oral admin. daily for 22 days (7 days after cell injection)	- No toxicity up to 100 mg/kg - Dose- and time-dependent decrease in tumor volume with a stronger effect for the 60 mg/kg dose. For 50% of the animals, tumor completely disappeared upon 35 day treatment - 25% decrease of phospho-cofiline for the dose 60 mg/kg	Rak et al., 2014 [63]
T56-LIMKi	Photothrombotic stroke	CD-1 mice 14–15 weeks photothrombotic stroke (PTS)	- 30 mg/kg in 0.5% CMC - Oral admin. daily for 5 days after PTS. 1st admin. 1 h upon PTS	- Reduction of the infarction volume by 2 or 3.4 times at 7 or 14 days after PTS - Rescued morphological changes in the brain - Reduced number of pathologically altered cells - Increased number of normochromic neurons	Demyanenko and Uzdensky 2021 [64]
Damnacanthal	Cutaneous immune response	BALB/c mice; ears painted with 2,4,6-trinitrochlorobenzene (TNCB)	- 20 µM 30 min. before TNCB treatment, immediately after and 12 h later - Topical admin.	- Suppressed hapten-induced migration of Langerhans cells in ears	Ohashi et al., 2014 [66]
CEL-Amide	Acute Myeloblastic Leukemia	FLT3-ITD+ NOD-SCID mice injected intravenously with MOLM-13-LUC	- 10 control, 10 treated - 10 mg/kg in 0.15 M NaCl - Oral admin. until death (11 days upon cell injection)	- Delay of MOLM-13-LUC engraftment for the combination of CEL-Amide and midostaurin	Djamai et al., 2021 [67]

## Data Availability

Not applicable.
